# Pharmaceutical Payments to Japanese Board‐Certified Head and Neck Surgeons Between 2016 and 2019

**DOI:** 10.1002/oto2.31

**Published:** 2023-02-17

**Authors:** Anju Murayama, Haruki Shigeta, Sae Kamamoto, Erika Yamashita, Hiroaki Saito, Toyoaki Sawano, Divya Bhandari, Sunil Shrestha, Eiji Kusumi, Tetsuya Tanimoto, Akihiko Ozaki

**Affiliations:** ^1^ Medical Governance Research Institute Minato‐ku Tokyo Japan; ^2^ School of Medicine Tohoku University Sendai city Miyagi Japan; ^3^ Faculty of Medicine Hamamatsu University School of Medicine Hamamatsu Shizuoka Japan; ^4^ Department of Internal Medicine Soma Central Hospital Soma City Fukushima Japan; ^5^ Department of Surgery Jyoban Hospital of Tokiwa Foundation Iwaki City Fukushima Japan; ^6^ School of Pharmacy, Monash University Malaysia Jalan Lagoon Selatan Bandar Sunway Jalan Lagoon Selatan Malaysia; ^7^ Department of Internal Medicine Navitas Clinic Shinjuku Shinjuku‐ku Tokyo Japan; ^8^ Department of Internal Medicine Navitas Clinic Tachikawa Tachikawa City Tokyo Japan; ^9^ Department of Breast and Thyroid Surgery Jyoban Hospital of Tokiwa Foundation Iwaki City Fukushima Japan

**Keywords:** conflict of interest, head and neck cancer, Japan, pharmaceutical payment

## Abstract

**Objective:**

To evaluate the magnitude, prevalence, and trend of the financial relationship between Japanese head and neck surgeons and pharmaceutical companies between 2016 and 2019.

**Study Design:**

Cross‐sectional analysis.

**Setting:**

Japan.

**Methods:**

This study evaluated personal payments concerning lecturing, consulting, and writing paid by 92 major pharmaceutical companies to all Japanese head and neck surgeons board‐certified by the Japan Society for Head and Neck Surgery between 2016 and 2019. The payments were descriptively analyzed and payment trend were assessed using population‐averaged generalized estimating equations. Further, the payments to board executive board members with specialist certification were also evaluated separately.

**Results:**

Of all 443 board‐certified head and neck surgeons in Japan, 365 (82.4%) received an average of $6443 (standard deviation: $12,875), while median payments were $2002 (interquartile ranges [IQR] $792‐$4802). Executive board specialists with a voting right received much higher personal payments (median $26,013, IQR $12,747‐$35,750) than the non‐executive specialists (median $1926, IQR $765‒$4134, *p* < .001) and the executive board specialists without a voting right (median $4411, IQR $963‐$5623, *p* = .015). The payments per specialist and prevalence of specialists with payments annually increased by 11.4% (95% CI: 5.8%‐17.2%; *p* < .001) and 7.3% (95% CI: 3.8%‐11.0%; *p* < .001), respectively.

**Conclusion:**

There were increasingly widespread and growing financial relationships with pharmaceutical companies among head and neck surgeons in Japan, alongside of introduction of novel drugs. The leading head and neck surgeons received much higher personal payments from pharmaceutical companies, and no sufficient regulation was implemented by the society in Japan.

The concern for the influence of physicians' financial conflicts of interest (COIs) with pharmaceutical companies on healthcare jeopardized trust in healthcare and evoked motivation demanding greater transparency in the relationships worldwide.^1^, ^2^ In Japan, all pharmaceutical companies belonging to the Japan Pharmaceutical Manufacturers Association (JPMA), the largest pharmaceutical trade organization in Japan, have been demanded to disclose their payments to physicians on their company webpage since 2013.^3^ This payment disclosure enabled publications of the financial relationships between physicians and pharmaceutical companies with detailed amounts of payments in several specialties.^4^, ^5^, ^6^, ^7^, ^8^, ^9^, ^10^

Among several specialties, we speculated head and neck surgeons (HNSs) have increasing financial ties with pharmaceutical companies. One of the main diseases in the specialty is head and neck cancers including oral cavity cancer, pharyngeal cancer, and thyroid cancer. While surgery and radiotherapy are the key treatment for head and neck cancers, chemotherapy has advanced dramatically over the past 2 decades.^11^ In 2017, nivolumab (OPDIVO®) was approved for head and neck cancers in Japan. Another immune checkpoint inhibitor, pembrolizumab (KEYTRUDA®) was approved for head and neck cancers in 2019. Considering the introduction of several novel drugs for head and neck cancers and the increased role of board‐certified HNSs, the HNSs were speculated to have substantial financial relationships with pharmaceutical companies.

HNSs' financial relationships with pharmaceutical companies in the United States (US) were well‐described in studies conducted on otolaryngologist‐head and neck surgeons since the launch of Open Payments Database.^12^, ^13^, ^14^, ^15^, ^16^ The Open Payments Database is the legal‐binding payment database including all financial transfers from pharmaceutical and medical devices companies to physicians. However, there was no study assessing financial relationships with pharmaceutical companies among the HNSs in Japan. This study aimed to elucidate the magnitude and trend of personal payments from pharmaceutical companies to the board‐certified HNSs through the recent years in Japan.

## Methods

### Study Design

This study is a cross‐sectional analysis evaluating the financial relationships between all board‐certified HNS specialists and pharmaceutical companies in Japan. All HNSs who were certified by the Japan Society of Head and Neck Surgery (JSHNS) were included in this study. The JSHNS, the sole and largest professional medical society for head and neck surgery in Japan, trains and certifies HNSs who have abundant skills and knowledge in head and neck surgery and can provide multidisciplinary treatment of head and neck cancers under the name of “Head and Neck Cancer Specialist.”

### Data Collection

HNSs' names and affiliation were extracted from the official JSHNS webpage (https://www.jshns.org/modules/list_specialist/index.php) on February 10, 2022. At the time of our data extraction, the name list of board‐certified HNSs was last updated on January 20, 2022. The name list of executive board members of the JSHNS between 2020 and 2021 were also collected from the JSHNS webpage (https://www.jshns.org/modules/about/index.php?content_id=6) and the affiliation and position of the board‐certified HNSs were manually collected online to evaluate the strength of financial relationships between pharmaceutical companies and HNSs with a leading role.^17^, ^18^, ^19^, ^20^ To evaluate the association between payment amounts and the number of drugs with new approval and additional indications for head and neck cancers and thyroid cancers, all drugs approved between 2011 and 2021 were extracted from the approval drug list issued by the Pharmaceuticals and Medical Devices Agency. The Pharmaceutical and Medical Devices Agency is the sole official agency reviewing and approving drugs in Japan, similar to the US Food and Drug Administration.^21^

The payments concerning lecturing, writing, and consulting paid to the HNSs were extracted from all pharmaceutical companies belonging to the JPMA between 2016 and 2019. The JPMA transparency guidance voluntarily demands all member companies to disclose their payments to healthcare professionals and organizations. This payment disclosure is self‐regulated by the pharmaceutical industry association and there is no penalty for deviation from the guidance, which is one of the major differences between the US Open Payments program and JPMA transparency guidance. As of February 2022, the payment data of 2019 were the latest analyzable data in Japan. The payments from a total of 92 pharmaceutical companies were included in this study. The pharmaceutical companies disclosed payments for lecturing, writing, and consulting on the basis of individual physicians, but smaller and more prevalent payment categories such as food and beverages, travel and accommodation fees, and reimbursement for trial enrollment were not individually disclosed by the companies, as we noted previously.^5^, ^7^, ^8^, ^22^ The extracted raw payment data were included as Supplemental Material 1.

### Analysis

First, we conducted descriptive analyses for payment data. Average and median values were reported based on only HNSs receiving payment in each year, as in other studies.^8^, ^15^, ^16^, ^23^, ^24^ Second, to evaluate payment concentration among the HNSs, the Gini index and the shares of the payment values per specialist were calculated, as performed previously^5^, ^8^, ^9^, ^25^, ^26^ Third, to evaluate the trend between affiliations and positions, we used the robust adjustment. We also observed the affiliations and positions of HNSs who received more than $1000 continuously for 4 years. Fourth, we descriptively calculated payments between the HNSs with nonboard membership, the executive board HNSs without a voting right, and executive board HNSs with a voting right. The difference of payments between the 3 groups were evaluated by the Kruskal‐Wallis *H* test, and then the differences between each 2 group were assessed by Mann‐Whitney *U* test with the Bonferroni correction, as the payments were not normally distributed. Fifth, to evaluate the payment trends between 2016 and 2019, the population‐averaged generalized estimating equation (GEE) was performed, using the panel data of the personal payments in each specialist. As the payment distribution was highly skewed (Supplemental Material 2) negative binomial GEE model for the payment values per HNS, and linear GEE log‐linked model with binomial distribution for the prevalence of HNSs with payments were selected. Because several pharmaceutical companies disaffiliated from the JPMA and newly joined the JPMA, there were several companies without payment data over the 4 years. Thus, the trends of payments were calculated based on payments from all data‐collected companies and companies with payment data for the 4 years, as in our previous studies.^6^, ^8^, ^9^, ^27^ Finally, we calculated Spearman's correlation between number of new approvals or additional indications for head and neck cancers and (1) 4‐years total payments and (2) number of HNSs with payments on the pharmaceutical company level. As the total payments and number of HNSs with payments were not normally distributed, Spearman's correlation was used. The payment values were converted from Japanese yen (¥) to US dollars ($) using 2019 average monthly exchange rates of ¥109.0 per $1.

### Ethical Approval

The Ethics Committee of the Medical Governance Research Institute approved this study. As this study was a cross‐sectional analysis of publicly available information, informed consent was waived by the Ethics Committee.

## Results

### Overview and Per‐Specialist Payments

A total of 443 HNSs were identified on the JSHNS webpage as of February 10, 2022. Of the 443 eligible board‐certified HNSs, 365 (82.4%) received at least 1 payment from 55 (60.0%) pharmaceutical companies between 2016 and 2019. Total payment amounts and number of instances were $2,351,621 and 3348 instances over the 4 years. The median was $2002 (interquartile range [IQR] $792‐$4802) in payments; 4.0 (IQR 2.0‐9.0) in payment instances; and 3.0 (IQR 2.0‐5.0) in number of pharmaceutical companies per specialist (Table 1).

**Table 1:** Summary of Personal Payments from Pharmaceutical Companies to Board‐Certified Head and Neck Surgery Specialists Between 2016 and 2019

Variables	
Total	
Payment values, $	2,351,621
Instances, n	3348
Companies, n	52
Average per specialist (SD)	
Payment values, $	6443 (12,875)
Instances, n	9.2 (13.9)
Companies, n	4.1 (3.8)
Median per specialist (IQR)	
Payment values, $	2002 (792‐4802)
Instances, n	4.0 (2.0‐9.0)
Companies, n	3.0 (2.0‐5.0)
Range	
Payment values, $	95‐102,113
Instances, n	1.0‐105
Companies, n	1.0‐23.0
Physicians with specific payments, n (%)	
Any payments	365 (82.4)
Payments > $500	320 (72.2)
Payments > $1000	254 (57.3)
Payments > $5000	88 (19.9)
Payments > $10,000	55 (12.4)
Payments > $50,000	10 (2.3)
Payments > $100,000	1 (0.23)
Gini index	0.764
Category of payments	
Lecturing	
Payment value, $ (%)	2,129,986 (90.6)
Instances, n (%)	3050 (91.1)
Consulting	
Payment value, $ (%)	106,786 (4.5)
Instances, n (%)	112 (3.3)
Writing	
Payment value, $ (%)	99,830 (5.1)
Instances, n (%)	1086 (5.0)
Other	
Payment value, $ (%)	15,020 (0.6)
Instances, n (%)	15 (0.4)

For the payment distribution, 72.2%, 57.3%, 19.9%, 12.4%, and 2.3% of HNSs received more than $500, $1000, $5000, $10,000, and $50,000, respectively (Supplemental Material 3). The Gini index for the 4‐year cumulative payments per HNS was 0.764. Top 1%, 5%, 10%, and 25% of HNSs occupied 14.1% (95% confidence interval (CI) 10.8%‐17.4%), 46.5% (95% CI 41.0%‐52.1%), 66.5% (95% CI 62.0%‐71.0%), and 84.6% (95% CI 81.8%‐87.5%) of total payments, respectively (Supplemental Material 3). The highest payment was $102,113. The most common payment category was lecturing and 80.1% (355) of HNSs received 1 or more lecturing payments over the 4 years.

### Personal Payments and the Physicians' Affiliations and Positions

Among 443 board‐certified HNSs, 221 (49.9%) worked at universities or university hospital (Supplemental material 4). Compared to university staff who are not professors, university professors significantly received higher per‐physician personal payments (relative monetary value: 9.1times [95% CI 6.5‐12.8], *p* < .001), while the proportion of HNSs receiving payments did not reach statistical significance (87.3% vs 94.6%, relative proportion: 1.1 [95% CI 0.996‐1.2], *p* = .06). 52 HNSs (11.7%) continuously received more than $1000 for the 4 years, and among them 31 (59.6%) were university professors (Supplemental material 5).

### Payments to the JSHNS Executive Board Members

We further investigated all 31 executive board members in 2021. Of 31 members, 18 (58.1%) were the board‐certified HNSs. All 18 executive board members with specialist certification accepted personal payments from pharmaceutical companies between 2016 and 2019 (Table 2). Moreover, there was a statistically significant higher payment among the executive board HNSs with a voting right (median $26,013 [IQR $12,747‐$35,750]) than the HNSs with nonboard membership (median $1926 [IQR $765‐$4134], *p* < .001) and the executive board HNSs without a voting right (median $4411 [IQR $963‐$5623], *p* = .015).

**Table 2:** Payments to the Board‐Certified Head and Neck Surgery Specialists by Japan Society of Head and Neck Surgery With the 2020–2021 Executive Board Membership from Pharmaceutical Companies Between 2016 and 2019

			Payments, $				
Executive board members	Position in the Society executive board^a^	Ranking^b^	2016	2017	2018	2019	Four‐years combined
A	Executive board director	2	6233	15,887	25,565	20,165	67,849
B	Executive board chairperson	3	13,037	26,395	15,715	11,512	66,658
C	Executive board secretary	13	2759	5211	9707	22,594	40,271
D	Executive board director	14	8272	13,278	11,541	7124	40,215
E	Executive board director	20	7077	6361	11,437	6410	31,285
F	Executive board director	23	8070	6812	12,404	2673	29,958
G	Executive board director	26	4965	5544	9560	7895	27,964
H	Executive board director	30	1737	6866	4391	11,068	24,063
I	Executive board director	35	4829	5501	4291	4496	19,116
J	Executive board director	42	1430	4189	3707	5770	15,097
K	Executive board director	51	4189	867	2657	2686	10,398
L	Executive board director	54	1941	0	2657	5708	10,306
M	Executive board director	70	550	1174	2043	2465	6233
N	Executive board secretary	76	826	1941	1173	1683	5623
O	Executive board secretary	91	0	2554	1072	1224	4851
P	Executive board secretary	108	511	1306	817	1338	3972
Q	Executive board secretary	256	0	275	275	413	963
R	Executive board secretary	310	0	0	511	0	511

^a^ Positions in the executive board of Japan Society of Head and Neck Surgery between 2020 and 2021.

^b^ Ranking among the 443 head and neck surgery specialists board‐certified by the Japan Society of Head and Neck Surgery.

### Payment Trend Between 2016 and 2019

The median annual payments per specialist increased from $817 (IQR $511‐$2248) in 2016 to $1027 (IQR $520‐$2284) in 2019, with an average annual change of 12.4% (95% CI 6.8%‐18.4%, *p* < .001) (Table 3). The number of HNSs receiving payments also annually increased from 189 (42.7%) in 2016 to 260 (58.7%) in 2018, while decreased to 245 (55.3%) in 2019. Increasing trend of number of HNSs receiving payments by 8.0% (95% CI 4.4%‐11.7%; *p* < .001) per year was observed. Limiting payments from 50 (90.9%) companies with 4‐years data, the payments per HNS and fraction of HNSs with payments also annually increased by 11.4% (95% CI 5.8%‐17.2%; *p* < .001) and 7.3% (95% CI 3.8%‐11.0%; *p* < .001), respectively. 20.3% to 29.1% of HNSs received more than $1000 per year and a total of 45.4% (201) of HNSs received more than $1000 per year at least 1 year.

**Table 3:** Trend of Personal Payments from Pharmaceutical Companies to Board‐Certified Head and Neck Surgery Specialists Between 2016 and 2019

Variables	2016	2017	2018	2019	Average yearly change (95% CI), %	*p* value	Combined total
All pharmaceutical companies							
Total payments, $	422,572	656,954	630,954	641,141	‒	‒	2,351,621
Average payments (SD), $	2236 (3172)	2760 (4728)	2427 (3968)	2617 (4311)	12.4 (6.8‐18.4)	<0.001	6443 (12,875)
Median payments (IQR), $	817 (511‐2248)	1022 (511‐2350)	970 (511‐2146)	1027 (520‐2284)			2002 (792‐4802)
Payment range, $	95‐17,160	138‐28,503	95‐25,565	102‐36,111	‒		95‐102,113
Physicians with specific payments, n (%)							
Any payments	189 (42.7)	238 (53.7)	260 (58.9)	245 (55.3)	8.0 (4.4‐11.7)	<0.001	365 (82.4)
Payments > $500	148 (33.4)	187 (42.2)	203 (45.8)	209 (47.2)	11.0 (6.6‐15.5)	<0.001	320 (72.2)
Payments > $1000	90 (20.3)	127 (28.7)	129 (29.1)	129 (29.1)	10.3 (4.8‐16.2)	<0.001	254 (57.3)
Payments > $5000	26 (5.9)	38 (8.6)	33 (7.4)	37 (8.3)	8.7 (−0.9‐19.3)	0.78	88 (19.9)
Payments > $10,000	8 (1.8)	17 (3.8)	17 (3.8)	15 (3.4)	15.9 (0.0‐34.2)	0.049	55 (12.4)
Payments > $50,000	0 (0.0)	0 (0.0)	0 (0.0)	0 (0.0)	No observation	‒	10 (2.3)
Payments > $100,000	0 (0.0)	0 (0.0)	0 (0.0)	0 (0.0)	No observation	‒	1 (0.23)
Gini index	0.836	0.813	0.789	0.794	‒	‒	0.764
Pharmaceutical companies with 4‐years payment data							
Total payments, $	419,932	655,933	625,448	619,380	‒	‒	2,320,693
Average payments (SD), $	2222 (3149)	2756 (4705)	2415 (3946)	2581 (4209)	11.4 (5.8‒17.2)	<0.001	6358 (12,702)
Median payments (IQR), $	817 (511‐2248)	1,022 (511‐2350)	970 (511‐2146)	1,025 (520‐2261)			1941 (765‐4802)
Payment range, $	95‐17,160	138‐28,503	95‐25,565	95‐32,024	‒		95‐95,983
Physicians with specific payments, n (%)							
Any payments	189 (42.7)	238 (53.7)	259 (58.5)	240 (54.2)	7.3 (3.8‐11.0)	<0.001	365 (82.4)
Payments > $500	147 (33.2)	187 (42.2)	202 (45.6)	204 (46.0)	10.3 (5.3‐14.8)	<0.001	316 (71.3)
Payments > $1,000	89 (20.1)	127 (28.7)	128 (28.9)	125 (28.2)	9.5 (4.1‐15.2)	<0.001	250 (56.4)
Payments > $5,000	26 (5.9)	38 (8.6)	33 (7.4)	36 (8.1)	7.8 (−1.7‐18.1)	0.11	87 (19.6)
Payments > $10,000	8 (1.8)	17 (3.8)	17 (3.8)	15 (3.4)	15.9 (0.0‒34.2)	0.49	55 (12.4)
Payments > $50,000	0 (0.0)	0 (0.0)	0 (0.0)	0 (0.0)	No observation	‒	10 (2.3)
Payments > $100,000	0 (0.0)	0 (0.0)	0 (0.0)	0 (0.0)	No observation	‒	0 (0.0)
Gini index	0.836	0.812	0.789	0.799	‒	‒	0.860

Abbreviations: IQR, interquartile range; SD, standard deviation; US, United States.

In a subgroup analysis on the HNSs with executive board memberships, this increasing trend of payment values and prevalence of were observed among the HNSs without executive board memberships in both of payment values (average annual change: 9.8% [95% CI 4.2%‐15.7%]; *p* < .001) and prevalence of HNSs with payments (average annual change: 7.5% [95% CI 3.7%‐11.5%]; *p* < .001). However, there were no increasing trend of payment values and prevalence of HNSs with payments among the executive board HNSs with a voting right.

### Payment by Pharmaceutical Companies

**Figure 1:**
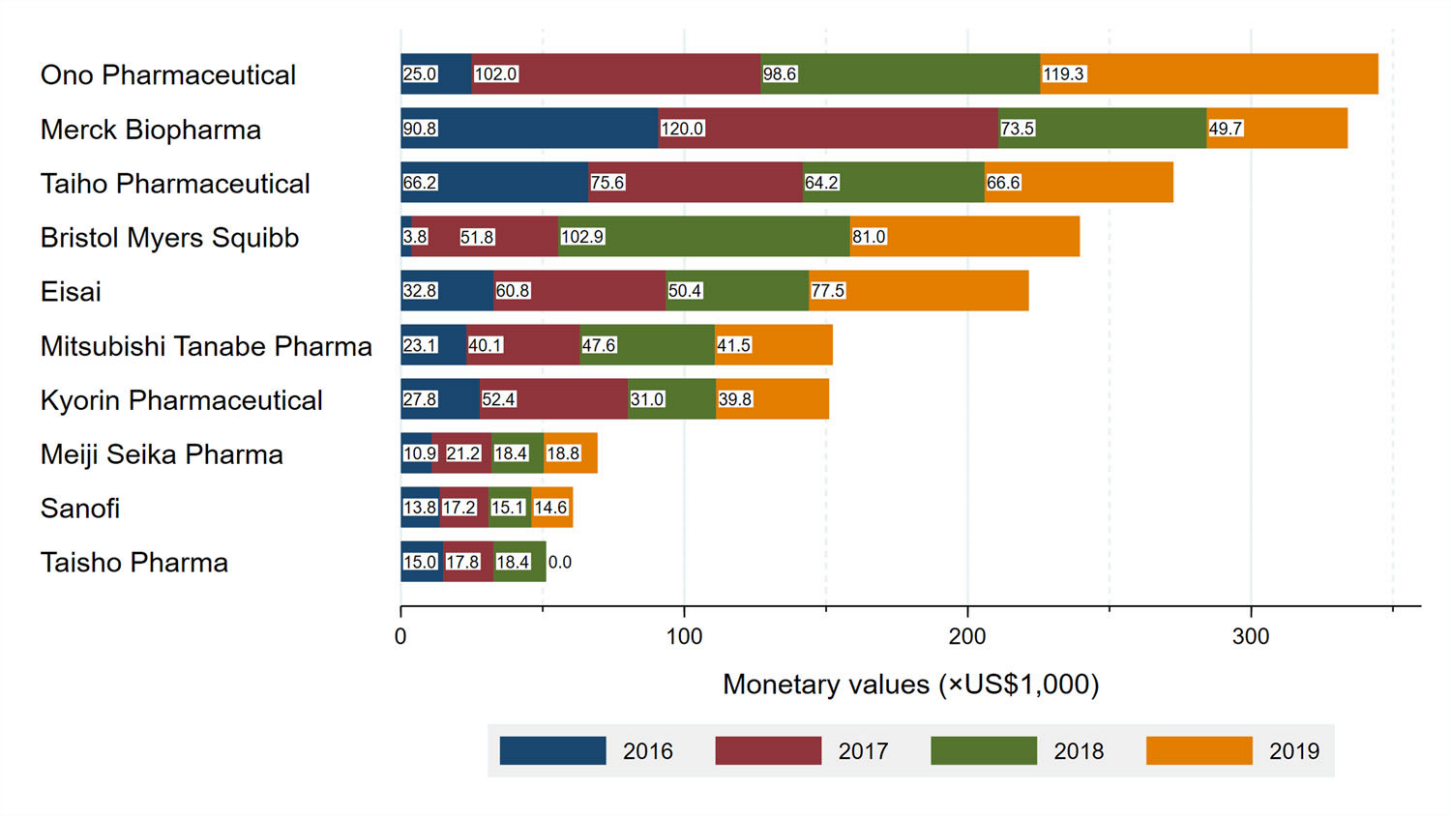
Payment trends by company.

**Figure 2:**
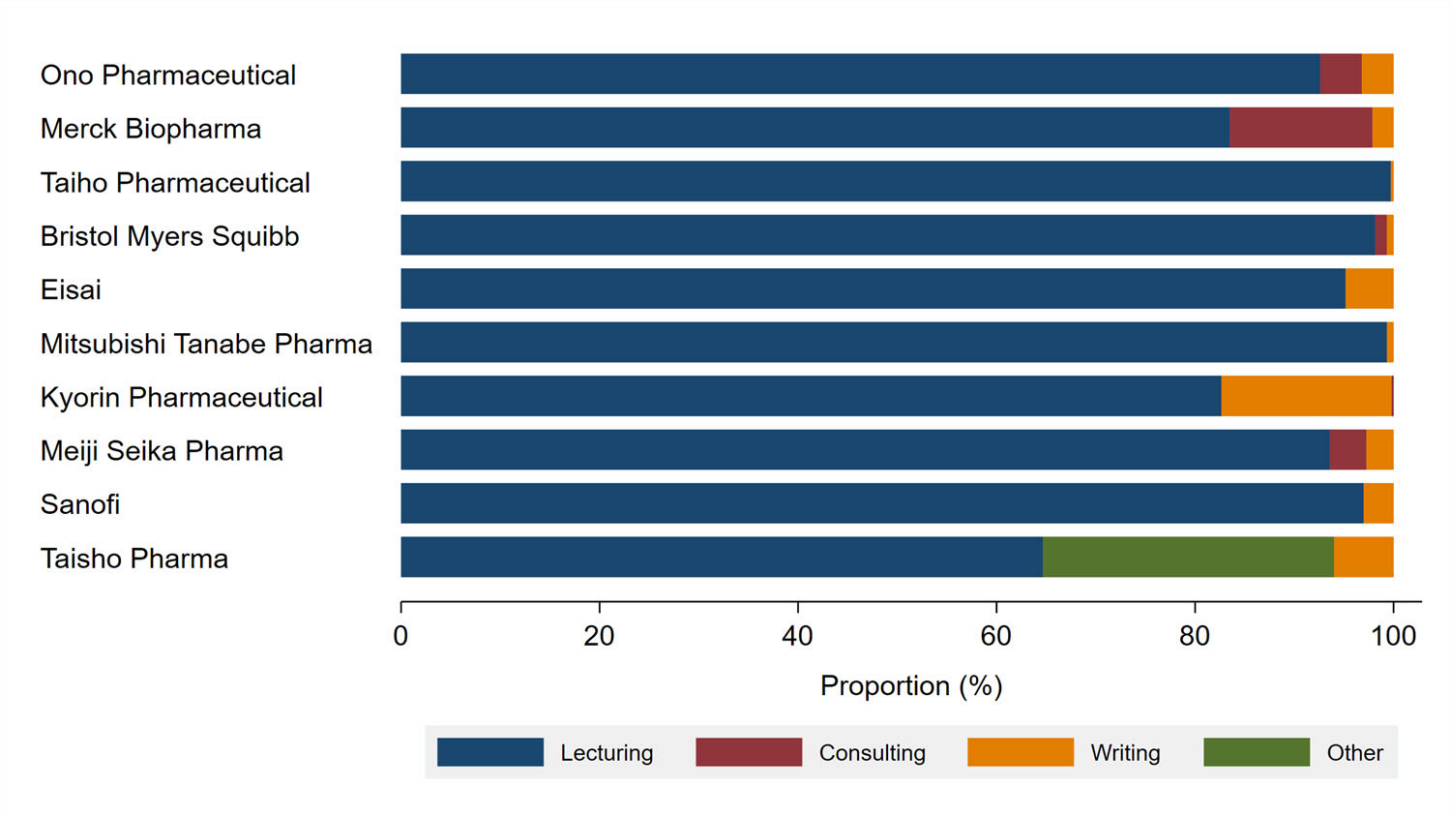
Payment categories by company.

Among the 92 pharmaceutical companies from which we collected data, 55 companies paid 1 or more payments to the board‐certified HNSs between 2016 and 2019. Payments from top 5 companies represented 60.1% ($1,412,381) of total payments, respectively. The largest payments were made by Ono Pharmaceutical ($344,844; 14.7%), followed by Merck Biopharma ($334,019, 14.2%), and Taiho Pharmaceutical ($272,528, 11.6%). Taiho Pharmaceutical distributed personal payments to the largest number of 165 HNSs, totaling 37.2% of all HNSs, followed by Merck Biopharma (160, 36.1%), Eisai (149, 33.6%). Payment trends and payment categories were described in Figures 1 and 2, respectively. There were 12 new or additional indications for head and neck cancers and thyroid cancers between 2011 and 2021 (Supplemental Material 6). Nine (75.0%) drugs were for chemotherapy and 2 (16.7%) drugs were for immunochemotherapy. Five (41.7%) drugs were approved for head and neck carcinoma, 5 (41.7%) drugs were approved for thyroid carcinoma, and remaining 2 (16.7%) drugs were approved for other cancers including parathyroid carcinoma and pituitary tumor. Rakuten Medical and Stella Pharma were not member companies of JPMA, so the 2 companies did not disclose payments to healthcare professionals in Japan.

There were moderate positive correlations between number of new or additional indications for head and neck cancers between 2011 and 2021 and the 4‐years total payments to the board‐certified HNSs (*r*(50) = 0.53; *p* < .001), as well as number of HNSs with payments (*r*(50) = 0.54, *p* < .001) in the Spearman's correlation.

## Discussion

This study found 82.4% of the Japanese board‐certified HNSs received a total of $2,351,621 in the personal payments for the reimbursement of lecturing, consulting, and writing between 2016 and 2019. However, only a very small proportion of these HNSs, such as the executive board members of the Japan Society for Head and Neck Surgery, received substantial personal payments from pharmaceutical companies. The substantial payments were often by the pharmaceutical companies which produced novel drugs for head and neck cancers. Our findings suggest several important similarities and differences from previous studies in the Japan and other developed countries.

First, surprisingly the Japanese HNSs, representing 4.8% of all Japanese otolaryngologists‐head and neck surgeons,^28^ received an average of $2236 to $2760 annual personal payments, and the median annual payments were $817 to $1027. One study reported that the average and median personal payments from pharmaceutical and medical device companies to the US otolaryngologists were $1096 and $169 in 2014, respectively.^14^ Comparing their findings, the Japanese HNSs received more than 5 times in median annual personal payments than those the US otolaryngologists received. Simply comparing the prevalence of physicians with payments, the percentage of the Japanese HNSs with payments (82.4%) was similar to that in the US otolaryngologists (84%‐86%) shown by the previous studies.^14^, ^15^, ^16^ However, our payment data only included payments for lecturing, consulting, and writing. Other common types of payments among otolaryngologists,^14^ such as meals, travel, and accommodations were not included. Despite these limited categories of payments, which would significantly underestimate the prevalence of HNSs receiving payments, as high as 82.4% of the Japanese HNSs were financially tied with pharmaceutical companies.

Furthermore, we found that the Japanese HNSs received as substantial personal payments as Japanese physicians in other specialties. We previously reported that the median personal payments from pharmaceutical companies between 2016 and 2019 were $596 (IQR $0‐$2436) in the pediatric hematologists/oncologists,^27^ $2210 ($715‐$8178) in the pulmonologists,^6^ and $2471 ($851‐$9677) in the hematologists.^9^ Furthermore these financial ties with pharmaceutical companies had become increasingly stronger and more prevalent.

The payment analysis on the company level has provided plausible reason for the increasing trends. We found positive associations between number of novel approved drugs and magnitude of personal payments to the HNSs, as well as the number of HNSs distributed payments. Payments from top 5 companies occupied for 60% of total payments, and among them, 4 companies had novel drugs for head and neck cancers. Among the 5 companies, Ono Pharmaceutical and Bristol Myers Squibb increasingly made personal payments to the HNSs mainly for lecturing since 2017. Ono Pharmaceutical paid 4.1 times higher payments in 2017, when Nivolumab was approved for head and neck cancer in Japan, compared to those in 2016 ($101,989 vs $25,033). Also, Bristol Myers Squibb made 13.7 times and surprisingly 27.2 times higher payments in 2017 ($51,787) and 2018 ($102,939) than those in 2016 ($3780), respectively.

Despite these increasing trend of payments as well as novel drugs for head and neck cancer, the JSHNS has not made any regulation for financial COI among the board‐certified HNSs. Accumulating evidence indicates financial COI influences physicians' clinical practice^29^ and this affects the otolaryngologists' prescriptions.^30^, ^31^ Given these situations, regulations of financial COI for the board‐certified HNSs are essential, such as limiting the maximum monetary value of personal payments from pharmaceutical companies. For example, the Danish Medicines Agency restricts all Danish medical physicians not to received more than DKK 200,000 (equal to about $30,000 and about one‐sixth of annual Danish physician salary) without permission from the Danish government.^32^

We also found that the leading HNSs in Japanese head and neck surgery such as the JSHNS executive board members received more substantial payments and they develop and implement regulations and statements for the HNSs endorsed by the JSHNS. Financial COI among the JSHNS executive board members were declared by each executive member, according to the JSHNS policy, but the COI information was not publicly disclosed due to the privacy of members. Financial independency from any entities and integrity to the patients are the most essential bases of all professional medical societies.^33^, ^34^ However, most of Japanese professional medical societies were more substantially tied with pharmaceutical companies compared to other developed countries. Saito et al found that 86.9% of executive board members of major professional medical societies received a median of $7486 personal payments in a single year.^20^ The prevalence of executive members with payments and median personal payments were much higher than those in Australia (24.4% and $9861 between October 2015 and April 2018),^19^ the United States (71.6% and $6026 between 2017 and 2019),^18^ and France (83% and about $4200 per year).^17^ This substantial concentration of payments to Japanese executive members could be due to less transparency in healthcare and immature discourse on financial COIs compared to other developed countries.^10^ We believe full transparency in the financial relationships with pharmaceutical companies should be implemented among the leading physicians with public and authoritative position such as society executive board members and board‐certified HNSs, as payment disclosure increases public trust^35^ and simultaneously patients desire physicians to disclose their financial relationships and to be free from financial ties with industries.^36^, ^37^ Uniform payment database should be developed and more transparent and rigorous COI policy should be implemented among leading physicians, constantly updating in accordance with public demands^10^, ^37^ and global standards.^38^, ^39^

This study included several limitations. First, there would be underestimated payments to the HNSs from nonmember companies of JPMA. However, as the member companies accounted for 80.8% of total pharmaceutical sales in Japan in 2018,^40^ the underestimation of payments could be minimized by including data from all member companies. Second, currently, pharmaceutical companies do not disclose other categories of payments such as meals, travel, and stock ownerships, according to the JPMA guidance.^41^ This could have led to underestimations of the extent and prevalence of overall financial relationships between HNSs and pharmaceutical industry. Third, this study included all HNSs as of February 2022, as the JSHNS did not disclose its list of HNSs for previous years. Therefore, there would have been some HNSs who did not hold the specialist certification. Fourth, the payment magnitude and trend may not be applicable to other countries.

In conclusion, the majority of the Japanese board‐certified HNSs were financially tied with pharmaceutical companies manufacturing novel drugs between 2016 and 2019. These financial ties became increasingly prevalent and strong in overall HNSs. Additionally, the HNSs in leading roles received much higher personal payments from pharmaceutical companies than those in other developed countries, and no sufficient regulation was implemented by the professional medical society in Japan.

## Author Contributions

**Anju Murayama**, data collection, study concept and design, resources, statistical analysis, drafting of the manuscript, reviewing of the manuscript, and study supervision; **Haruki Shigeta**, data collection, study concept and design, and drafting of the manuscript; **Sae Kamamoto**, data collection, study concept and design, and drafting of the manuscript; **Erika Yamashita**, data collection, reviewing of the manuscript; **Hiroaki Saito**, study concept and design, statistical analysis, drafting of the manuscript, and reviewing of the manuscript; **Toyoaki Sawano**, study concept and design, and reviewing of the manuscript; **Divya Bhandari**, study concept and design, and critically reviewing of the manuscript; **Sunil Shrestha**, study concept and design, and critically reviewing of the manuscript; **Eiji Kusumi**, study concept and design, and critically reviewing of the manuscript; **Tetsuya Tanimoto**, study concept and design, drafting of the manuscript, and study supervision; **Akihiko Ozaki**, study concept and design, data analysis, drafting of the manuscript, and study supervision; all authors had full access to all the data in the study and take responsibility for the integrity of the data and the accuracy of the data analysis.

## Disclosures

### Competing interests

Dr Saito received personal fees from TAIHO Pharmaceutical Co., Ltd. outside the scope of the submitted work. Dr Kusumi received personal fees from Otsuka Pharmaceutical outside the scope of the submitted work. Drs Ozaki and Tanimoto received personal fees from Medical Network Systems, a dispensing pharmacy, outside the scope of the submitted work. Dr Tanimoto also received personal fees from Bionics Co., Ltd., a medical device company, outside the scope of the submitted work. Regarding nonfinancial conflicts of interest among the study authors, all are engaged in ongoing research examining financial and nonfinancial conflicts of interest among healthcare professionals and pharmaceutical companies in Japan. Individually, Anju Murayama, Hiroaki Saito, Toyoaki Sawano, Tetsuya Tanimoto, and Akihiko Ozaki have contributed to several published studies addressing conflicts of interest and quality of evidence among clinical practice guideline authors in Japan and the United States. The other authors have no example conflicts of interest to disclose.

### Sponsorships

This study was funded in part by the Medical Governance Research Institute. This non‐profit enterprise receives donations from a dispensing pharmacy, namely Ain Pharmacies, Inc., other organizations, and private individuals.

### Funding sources

This study received support from the Tansa (formerly known as the Waseda Chronicle), an independent non‐profit news organization dedicated to investigative journalism. None of the entities providing financial support for this study contributed to the design, execution, data analyses, or interpretation of study findings and the drafting of this manuscript.

## Supporting information

Supplemental Material 1. Anonymized raw payment dataset.

Supplemental Material 2. Distribution of payment values per specialist.

Supplemental Material 3. Payment concentration.

Supporting information.

Supporting information.

Supporting information.
