# Supplementary figures and images for: Pharmaceutical Payments to Japanese Board‐Certified Head and Neck Surgeons Between 2016 and 2019
Source: OTO Open. 2023 Feb 17;7(1):e31. doi: 10.1002/oto2.31 (PMC10046701; doi:10.1002/oto2.31)

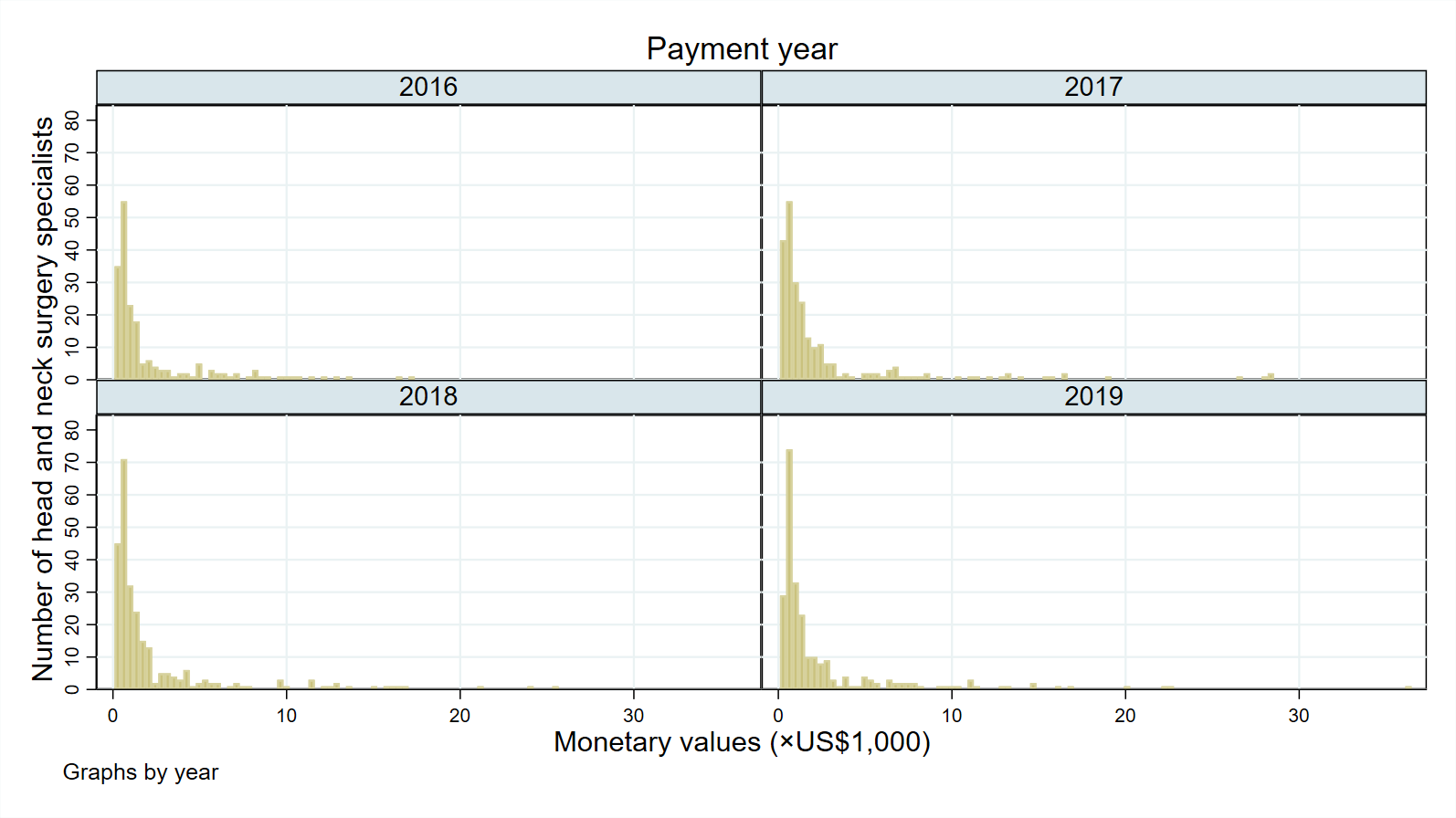

Supplement: Supplementary file 2 — Supplemental Material 2. Distribution of payment values per specialist. [file OTO2-7-e31-s005.png]

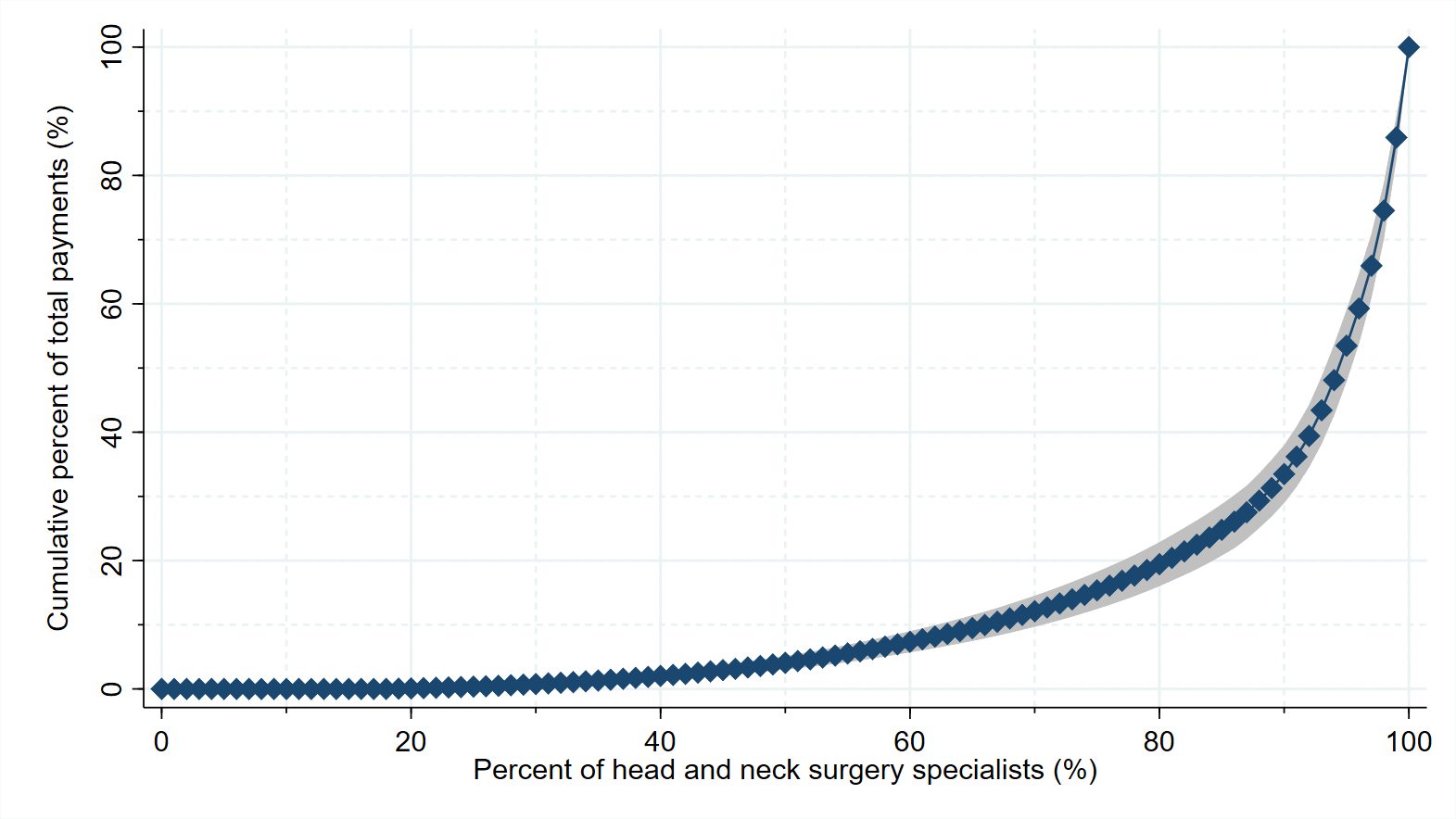

Supplement: Supplementary file 3 — Supplemental Material 3. Payment concentration. [file OTO2-7-e31-s006.png]
