# Supplementary material for: Pharmaceutical Payments to Japanese Board‐Certified Head and Neck Surgeons Between 2016 and 2019
Source: OTO Open. 2023 Feb 17;7(1):e31. doi: 10.1002/oto2.31 (PMC10046701; doi:10.1002/oto2.31)
Supplement: Supplementary file 4 — Supporting information. [file OTO2-7-e31-s002.docx]

Supplemental material 4. Personal payments from pharmaceutical companies to the board-certified head and neck surgery specialists between 2016 and 2019 by affiliation and position

| Affiliation and Position | Number of physicians,  n (%) | Physicians with payments,  n (%) ^a^ | Total payments, $ | Median payments (IQR), $ | Payment range, $ | Relative payments (95% CI) | |
| --- | --- | --- | --- | --- | --- | --- | --- |
|  |  |  |  |  |  | Relative proportion of physicians with payments | Relative monetary value |
| Non-professor at university/university hospital | 165 (37.3) | 144 (87.3) | 427,769 | 1,753 (740‒3,491) | 102‒25,318 | Ref. | Ref. |
| Professor at university/university hospital | 56 (12.7) | 53 (94.6) | 1,323,533 | 17,914 (4,674‒36,068) | 307‒102,113 | 1.1 (1.0‒1.2) | 9.1 (6.5‒12.8)^***^ |
| National/Prefectural/City　hospital | 108 (24.4) | 79 (73.1) | 346,602 | 1,644 (715‒3,983) | 275‒32,705 | 0.84 (0.74‒0.95)^**^ | 1.2 (0.8‒1.9) |
| Private hospital | 89 (20.1) | 69 (77.5) | 214,733 | 1,646 (715‒2,835) | 95‒40,271 | 0.89 (0.78‒1.0) | 0.93 (0.55‒1.6) |
| Clinic | 24 (5.4) | 19 (79.2) | 37,962 | 1,694 (409‒3,306) | 284‒5,304 | 0.91 (0.73‒1.1) | 0.61 (0.38‒0.97)^*^ |
| Pharmaceutical company | 1 (0.2) | 1 (100) | 1,022 | 1,022 (1,022‒1,022) | 1,022‒1,022 | ‒ | ‒ |

Abbreviations: interquartile range (IQR); 95% confidence interval (95% CI)

*p<0.05, **p<0.01, ***p<0.001

^a^ Chi-square test was performed.

Japanese yen (¥) was converted into US dollars using the 2019 average monthly exchange rates of ¥109.0 per $1

Pharmaceutical company was not included because the observation was too small to estimate the relative payments.
