# Supplementary material for: Pharmaceutical Payments to Japanese Board‐Certified Head and Neck Surgeons Between 2016 and 2019
Source: OTO Open. 2023 Feb 17;7(1):e31. doi: 10.1002/oto2.31 (PMC10046701; doi:10.1002/oto2.31)
Supplement: Supplementary file 5 — Supporting information. [file OTO2-7-e31-s001.docx]

Supplemental material 5. Affiliations and positions of board-certified head and neck surgery specialists who received more than $1,000 each year between 2016 and 2019

|  | Physicians who received payments  more than $1,000 each year, n (%) |
| --- | --- |
| **Affiliation and Position** | 52 (11.7) |
| Non-professor at university/university hospital | 7 (13.5) |
| Professor at university/university hospital | 31 (59.6) |
| National/Prefectural/City hospital | 10 (9.3) |
| Private hospital | 4 (4.5) |

Japanese yen (¥) was converted into US dollars using the 2019 average monthly exchange rates of ¥109.0 per $1.
