# Supplementary material for: Pharmaceutical Payments to Japanese Board‐Certified Head and Neck Surgeons Between 2016 and 2019
Source: OTO Open. 2023 Feb 17;7(1):e31. doi: 10.1002/oto2.31 (PMC10046701; doi:10.1002/oto2.31)
Supplement: Supplementary file 6 — Supporting information. [file OTO2-7-e31-s004.docx]

Supplemental Material 6. New approvals and additional indications for head and neck cancers and thyroid cancers between 2011 and 2021 in Japan

| Brand name | Name | Pharmaceutical companies | Approval date | Price per drug unit, $ | Indication | Category |
| --- | --- | --- | --- | --- | --- | --- |
| SOMATULINE | Lanreotide acetate | Manufacturer and distributor: Teijin Pharma | December 25, 2020 | 1,592 (60 mg for subcutaneous injection)  2,221 (90 mg for subcutaneous injection)  2,845 (120 mg for subcutaneous injection) | Thyroid-stimulating hormone secreting pituitary tumor | Additional indication |
| AKALUX | Cetuximab sarotalocan | Manufacturer and distributor: Rakuten Medical | September 25, 2020 | 9,420 (infusion 250 mg) | Unresectable locally advanced or locally recurrent head and neck cancers | New conditional approval |
| STEBORONINE | Boronophenylalanine B-10 | Manufacturer and distributor: Stella Pharma | March 25, 2020 | 4,075 (infusion 9000 mg/300 mL) | Unresectable locally advanced or locally recurrent head and neck cancers | New approval |
| KEYTRUDA | Pembrolizumab | Manufacturer and distributor: MSD | December 20, 2019 | 1,968 (injection 100 mg) | Recurrent or metastatic head and neck cancers | Additional indication |
| OPDIVO | Nivolumab | Manufacturer and distributor: Ono Pharmaceutical  Promotional partner: Bristol Myers Squibb | March 24, 2017 | 1,423 (intravenous infusion 100 mg)  293 (intravenous infusion 20 mg) | Recurrent or metastatic head and neck cancers | Additional indication |
| NEXAVAR | Sorafenib tosilate | Manufacturer and distributor: Bayer | February 29, 2016 | 44 (tables 200 mg) | Unresectable thyroid cancer | Additional indication |
| CAPRELSA | Vandetanib | Manufacturer and distributor: AstraZeneca (until July 2016) and Sanofi | September 28, 2015 | 72 (tablets 100 mg) | Unresectable medullary thyroid cancer | New approval |
| LENVIMA | Lenvatinib mesilate | Manufacturer and distributor: Eisai Promotional partner: MSD | March 26, 2015 | 37 (capsule 4 mg)  87 (capsule 10 mg) | Unresectable advanced or recurrent thyroid cancer | New approval |
| NEXAVAR | Sorafenib tosilate | Manufacturer and distributor: Bayer | June 20, 2014 | 44 (tablets 200 mg) | Unresectable differentiated thyroid carcinoma | Additional indication |
| REGPARA | Cinacalcet hydrochloride | Manufacturer and distributor: Kyowa Kirin | February 21, 2014 | 553 (tables 25 mg)  1,020 (tables 75 mg) | Hypercalcemia in patients with parathyroid carcinoma, and hypercalcemia in patients with primary hyperparathyroidism (HPT) who are unable to undergo parathyroidectomy or who experience recurrent primary HPT after the surgery | Additional indication |
| ERBITUX | Cetuximab | Manufacturer and distributor: Merck Biopharma Promotional partner: Bristol Myers Squibb (until April 2015) | December 21, 2012 | 337 (injection 100 mg) | Head and neck cancers | Additional indication |
| THYROGEN | Thyrotropin human alfa | Manufacturer and distributor: Sanofi | May 25, 2012 | 989 (intramuscular injection 0.9 mg) | Adjunctive treatment for radioiodine ablation of thyroid tissue remnants in patients who have undergone a near-total or total thyroidectomy for well-differentiated thyroid cancer and who do not have evidence of distant metastatic thyroid cancer | Additional indication |
